# Softening Effects in Biological Tissues and NiTi Knitwear during Cyclic Loading

**DOI:** 10.3390/ma14216256

**Published:** 2021-10-21

**Authors:** Yuri F. Yasenchuk, Ekaterina S. Marchenko, Sergey V. Gunter, Gulsharat A. Baigonakova, Oleg V. Kokorev, Alex A. Volinsky, Evgeny B. Topolnitsky

**Affiliations:** 1 Laboratory of Superelastic Biointerfaces, National Research Tomsk State University, 634050 Tomsk, Russia; yuri.yasenchuk@mail.tsu.ru (Y.F.Y.); mes@mail.tsu.ru (E.S.M.); guntersv@mail.tsu.ru (S.V.G.); baigonakova@mail.tsu.ru (G.A.B.); kokorevov@mail.tsu.ru (O.V.K.); 2 Department of Thoracic Surgery, Siberian State Medical University, 2 Moskovsky Trakt, 634055 Tomsk, Russia; e_topolnitskiy@mail.ru; 3 Department of Mechanical Engineering, University of South Florida, Tampa, FL 33620, USA

**Keywords:** titanium nickelide wire, knitted mesh, stress-strain curves, superelasticity, biological tissues

## Abstract

Samples of skin, tendons, muscles, and knitwear composed of NiTi wire are studied by uniaxial cyclic tension and stretching to rupture. The metal knitted mesh behaves similar to a superelastic material when stretched, similar to soft biological tissues. The superelasticity effect was found in NiTi wire, but not in the mesh composed of it. A softening effect similar to biological tissues is observed during the cyclic stretching of the mesh. The mechanical behavior of the NiTi mesh is similar to the biomechanical behavior of biological tissues. The discovered superelastic effects allow developing criteria for the selection and evaluation of mesh materials composed of titanium nickelide for soft tissue reconstructive surgery.

## 1. Introduction

Implant biocompatibility is very important since the number of implant surgeries is rapidly growing in various medical fields. At the same time, the variety of implant designs and the number of new materials used for tissues reconstruction is rapidly increasing. From the standpoint of materials science, biological tissues are composite materials containing biopolymers, liquids, and crystalline ceramics. The biomechanical behavior of biological tissues is diverse due to their different internal structures. Multiple reports have studied the deformation of blood vessels, muscle and connective tissues, skin, bones, and other types of biological tissues [1,2]. The deformation behavior of biological materials cannot be described using a universal micromechanical model. The mechanical behavior of biological tissues is described using multiple characteristics, including stiffness, elastic modulus, flow stress, yield strength, viscosity, and stress relaxation characteristics [3,4,5,6].

Modern implants are made from polymers, ceramics, metals, and composite materials, which have different structures and properties [7,8]. Mesh implants, stents, and blood vessel filters are widely used in vascular surgery. Thus, methods for calculating stress and strain distribution in implants and at the interface between metal mesh structures with the soft tissues have been developed [9]. For example, when installing mesh structures during hernia reconstructive surgery, it is necessary to take into account the long-term interaction of the implant with soft tissues. An incorrect assessment of the force interactions between the implant and biological tissues leads to implant failure or complications in the form of tissue and organ perforations, stenosis, scarring, damage, and the functional disruption of the adjacent organs. Currently, there are no objective assessment tools for the biomechanical compatibility of implants and biological tissues, which could be used by implant manufacturers and medical professionals. The development of objective criteria will help improve indications for implant use and their efficacy.

To study the rheology of biological tissues, different loading methods are used, and the following types of deformation are distinguished: tension, shear, hydrostatic compression, torsion, and bending. Unfortunately, no single methodology allows for an objective and detailed characterization of biomechanical properties of tissues in vivo. Currently, the description of biomechanical properties depends on the measurement method used. For example, to characterize the mechanical properties of skin, it is deformed using a fixed load, and then the strain and recovery are analyzed, which depend on skin density, stiffness, and elastic modulus [10].

Installed implants interact with various types of biological tissues. Biomaterials and biological tissue deformation behavior cannot be described using a universal micromechanical model. Taking into account biophysical and physical-mechanical differences at the biointerface, the degree of biomechanical compatibility of the implant and biological tissues can be assessed by comparing features of tensile and cyclic loading stress-strain curves. Some experts believe that such a comparative assessment is unacceptable due to large structural differences and physical mechanisms of biological tissues and biomaterial deformation. However, for a comparative assessment, differences in the structure of the implant material and biological tissues do not matter. This assessment should only take into account the similarities and differences of the stress-strain curves. These features can be successfully used for the selection of specific biomaterials for reconstructive surgery of specific soft tissues, based on their deformation characteristics obtained using a single technique and the same type of equipment in the range of physiological loads. It should be noted that the generalized data obtained from the literature are suitable for such a comparative methodology only at the initial planning research stage.

Vibrations and ultrasound methods are often used to study the viscous properties of polymers [11]. However, uniaxial tension remains one of the main methods for characterizing the mechanical response of biological tissues, superelastic materials, and shape memory alloys. The stress-strain curve describes the stresses that arise in the material in response to a deformation caused by uniaxial tension. The uniaxial tension of implant materials allows studying their mechanical response in comparison with biological tissues using unified methodological approaches.

The idea of biomechanical compatibility between an implant and biological tissue has existed for more than half a century, but the criteria for biomechanical similarity have not yet been developed. Modern implants are complex structures that consist of metals, ceramics, and polymers. Traditional implant materials composed of stainless steel, titanium, and ceramics do not have biomechanical compatibility with biological tissues under dynamic loading conditions [12,13]. Only polymers can deform similar to biological tissues [14,15,16]. The mechanics of hyperelastic polymer implants are closer than other materials to the biomechanics of superelastic soft tissues in the range of physiological loads and deformations. Therefore, the deformation of their interface occurs according to the same rheological laws.

Titanium nickelide (NiTi) superelastic alloys are widely used in cardiac and abdominal surgery, dentistry, traumatology, and gynecology [17,18,19]. Superelastic implants are actively used in the form of stents in endovascular surgery, orthodontic archwires in orthodontics, and metal-knitted materials in hernia restorations.

The viscoelastic behavior of NiTi alloys has been well studied within the framework of the theory of elasticity and martensitic transformations [20,21,22,23,24]. An external tensile load causes a direct martensitic transformation in NiTi implants accompanied by an increase in entropy and large heat losses in the entire sample volume due to friction between the austenite and martensite crystals. When describing the martensitic transformations induced by uniaxial tension, the Clausius–Clapeyron equation is used, which is derived from the equality of the thermodynamic potentials of the Gibbs free energy of the austenite and martensite phases [25]:

(1) $$\frac{d\sigma }{dT}=-\frac{\Delta S}{\varepsilon_{M}}=-\frac{\Delta H}{T_{0}\varepsilon_{M}}$$

Here, *dσ* is the stress change, *dT* is the change in the transition temperature when the stress changes by *dσ*, Δ*S* is the change in the entropy of transformation, Δ*H* is the latent heat of transition, *T*_0_ is the phase equilibrium temperature, and *ε_M_* is the martensitic strain.

However, this knowledge is difficult to apply to analyze the joint deformation behavior of superelastic implants and biological tissues using a unified methodological approach. The functioning of the interface between hyperelastic biological tissue and a superelastic implant is complex; therefore, the principles of its biomechanical analysis have not yet been developed.

The difficulty in selecting a superelastic alloy for a specific biological tissue is due to reversible martensitic transformation during deformation, which provides high NiTi endurance, caused by certain martensitic shear stress. The stress is caused by the load from the biological tissue in contact with the implant. Thus, it is necessary to calculate the martensitic shear stresses, which should be within the physiological load range of a particular biological tissue in contact with the implant. In this case, the calculated martensitic deformation should coincide with the physiological deformation of the contacting biological tissue. Such calculations have not yet been carried out.

Studies of the rubber-like behavior of pressed NiTi springs are of great interest [26,27]. Products composed of superelastic wire exhibit softening effects. The authors explain such changes using the elastic strengthening of the wire turns and the appearance of numerous contacts between the turns. Successful practical applications of superelastic implants around the world are based on a large number of tests of each design and the high qualifications of the developers. The wider application of this type of material requires the development of objective biomechanical criteria for the use of superelastic structures. The absence of such criteria creates increased application risks that limit the use of superelastic NiTi implants.

In this work, the mesh implants made from superelastic NiTi 40–100 µm diameter wire are studied. This type of implant is promising for bone and soft tissue reconstructive surgery. A unified approach to the rheological assessment of biological tissue and mesh implant during stretching makes it possible to develop simple and practical criteria for assessing the deformation superelastic behavior of an implant and biological tissue to increase the predictability of organ-preserving plastic surgery of soft biological tissues. Comparisons of the features of the engineering stress-strain curves of mesh implants and biological tissues under tensile and cyclic loads are a starting point for the selection criteria development. In further research, these stress-strain curves need to be used to obtain design models and parameters for the objective selection criteria.

## 2. Materials and Methods

**Figure 1:**
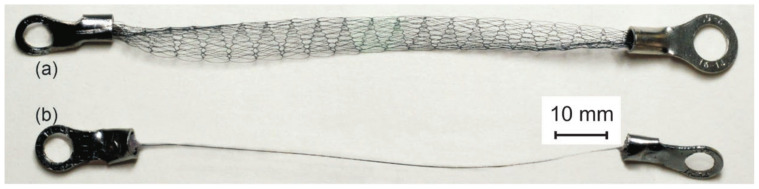
(**a**) NiTi knitted mesh and (**b**) NiTi superelastic wire.

To study the viscoelastic properties of knitted mesh, samples made from superelastic titanium nickelide 60 µm diameter wire and knitted NiTi mesh from 40 µm, 60 µm, and 90 µm diameter wires were prepared (Figure 1). Samples of knitted NiTi mesh and wire 100 mm long were pressed into grips with holes.

The wire with 40 µm, 60 µm, and 90 µm diameter was obtained from 20 mm × 240 mm ingots by thermomechanical treatment with intermediate annealing in 4 stages:

1. Rolling of the 20 mm diameter ingot to a 7 mm thick bar (20 cycles);
2. Rotary forging of the bar from 7 mm to 3.5 mm thickness (7 cycles);
3. Cold drawing of wire from 3.5 mm to 500 µm diameter (25 cycles);
4. Hot drawing of wire from 500 µm to 90–40 µm diameter (50–70 cycles).

**Figure 2:**
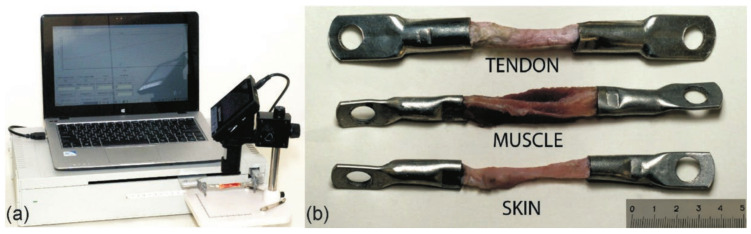
(**a**) Universal tensile testing machine and (**b**) soft biological tissues samples.

A knitted mesh with 50 loops width was obtained from the 40 µm, 60 µm and 90 µm diameter wires, annealed and deformed by rolling into a double ribbon at 500 °C. Samples 70 mm long were cut from the continuous mesh, and their ends were pressed into the grips. Samples of skin, tendons, and muscles 50 mm long were prepared from a fresh bovine knee joint. The ends of the samples were dehydrated in alcohol and formalin and pressed into metal grips (Figure 2).

Uniaxial stress-strain curves of viscoelastic materials were obtained using a custom-built software-controlled cyclic electromechanical universal tensile testing machine with a 2 kg maximum load. The tension was controlled using a personal computer, which allowed assigning and changing the number of cycles, strain rate, and strain limits. The tensile machine was equipped with universal sample grips fixed with screws. The tensile machine had a 3 μm displacement resolution and 0.004 N force resolution.

Transmission electron microscopy (TEM) observations of wire samples in a dark field and nano diffraction modes were carried using thin foils in the JEOL JEM-2100 microscope operated at an accelerating voltage of 200 kV. Thin foils were prepared from parallel sections using the FISCHONE INSTRUMENTS model 1051 TEM mill. Dimples in the samples were polished using the FISCHONE INSTRUMENTS model 200 dimpling grinder. Surface images of the superelastic NiTi wire were obtained using TESCAN MIRA3 LMU scanning electron microscope (SEM, 20 kV). Images of a knitted mesh made from 60 μm NiTi wire integrated into biological tissues were obtained using confocal laser scanning microscopy (ZEISS LSM 780 NLO).

Male Wistar rats weighing 160-180 g were obtained from the nursery of the Experimental Laboratory of Biomedical Technologies, Tomsk Research Institute of Balneology and Physiotherapy, Siberian Federal Research and Clinical Center, Federal Medical-Biological Agency of Russia. In the study, 12 male 10 weeks old inbred Wistar rats were randomly selected. All the animals were accommodated in cages with sterilized wood shavings as bedding material and acclimatized to standard laboratory conditions (21–22 °C, 40–50% humidity, 12 h light-dark cycle), and they were preoperatively denied food 24 h before surgery. Standard dietary patterns and water were provided ad-lib. The study using animals was conducted in accordance with the rules of laboratory practices in the Russian Federation (Order of the Ministry of Health and Social Development of the Russian Federation No. 199n, dated 4 January 2016).

All procedures using animals were carefully carried out, with strict adherence to the European Convention for the Protection of Vertebrate Animals used for Experimental and other Scientific Purposes (Strasburg, 1986), and with the European Communities Council Directive 86/609/EEC. All manipulations were undertaken under general ketamine anesthesia (1 mg/10 g dose). The study protocol was officially approved (approval code number №20/1116/2017) by the Bioethical Committee of Tomsk State University.

To study titanium nickelide mesh implant integration, experiments were performed using 12 adult Wistar rats. All manipulations and removal of animals from the experiments were carried out under general anesthesia. Preparation for surgery, anesthetic management, and management of the postoperative period were the same in all animals. An extensive post-resection defect of the anterior abdominal wall was modeled under general anesthesia and surgically replaced with a knitted titanium nickelide implant. The investigated implants were made from titanium nickelide 60 μm wire knitted mesh.

The animals underwent a 3–4 cm incision along the midline of the body on the anterior abdominal wall with a transition to the chest wall. The skin and subcutaneous tissue were mobilized, and the muscular–fascial and aponeurotic flap of the anterior abdominal wall with the xiphoid process was resected extrapleurally and extraperitoneally. A post-resection 2 cm × 3 cm defect was formed. The endoprosthesis was cut out according to the shape of the defect with an edge allowance. The endoprosthesis was fixed along the perimeter using polypropylene monofilaments 4/0 with interrupted or continuous sutures, capturing the edge of the implant into the sutures. The implant was fixed to the chest wall and diaphragm at the level of the resected xiphoid process. The surgical incisions were sutured in layers. The animals were removed from the experiment on days 15, 30, 60, and 90 after surgery.

## 3. Results and Discussion

Most soft biological tissues are deformed nonlinearly with a stiffening effect under uniaxial tension [28]. This nonlinearity depends on the strain and the strain rate. Soft biological tissues are classified as superelastic materials, which are characterized by a wide range of reversible elastic deformation reaching 10–500% [29,30,31]. Three regions of the stress-strain curve can be distinguished as the initial linear region with a low elastic modulus; the middle transition region of nonlinear deformation; the final linear region with a high modulus of elasticity.

**Figure 3:**
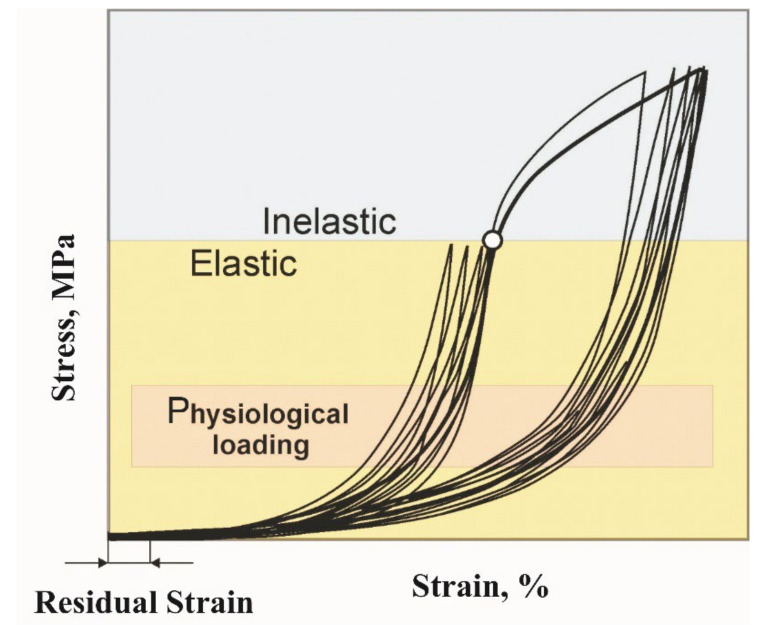
Generalized stress-strain curves of biological tissues showing physiological loading range.

The physiological loading range usually does not exceed 20% of the ultimate strength. As a rule, the physiological strain range is within 20–50% of the maximum strain and is located at the end of the low elastic modulus region or the beginning of the high elastic modulus region (Figure 3). Such generalizations cannot be extended to all biological tissues because their structure and biomechanical properties are extremely diverse. An implant for a certain biological tissue is designed to work in a certain range of physiological loading, while its ultimate strength should be significantly higher.

**Figure 4:**
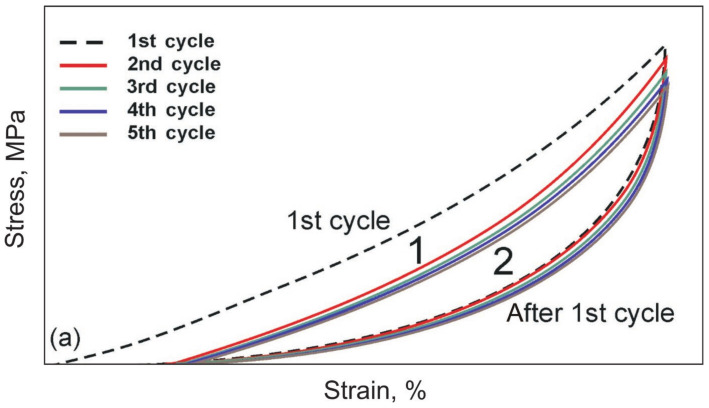

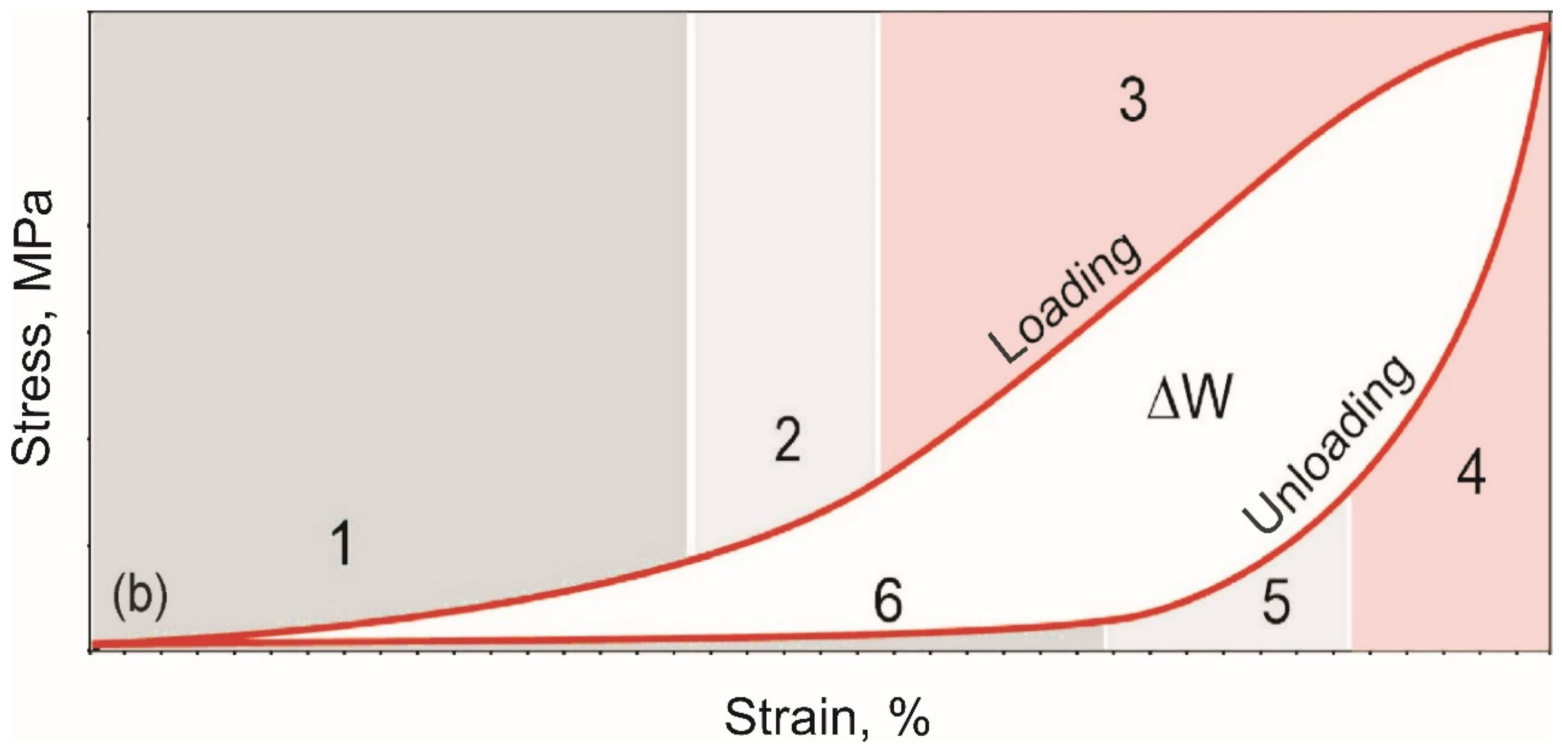
Schematic stress-strain curves of cyclic reversible tension of superelastic materials and biological tissues: (**a**) Mullins effect under cyclic tension; (**b**) single cycle with different deformation regions: 1—the initial linear region with low elastic modulus; 2 and 5—middle transition region of nonlinear deformation, 3—the final linear region with high elastic modulus, 4—elastic unloading region, 6—viscous unloading region. ΔW is the energy dissipated in one loading-unloading cycle, which is the difference between the areas under the loading and unloading curves.

Superelastic materials are characterized by stress hysteresis and the softening effect under cyclic loading, which is called the Mullins effect in polymers [32,33,34] (Figure 4). The hysteresis after the 1st cycle was significantly reduced, so it is convenient to highlight the hysteresis of the 1st and 2nd cycles. These effects were caused by the losses from the counteraction of viscous forces of internal friction and elastic forces. Such stress-strain curves are typical for soft biological tissues, as well as for filled rubbers and low modulus rubber-like materials.

**Figure 5:**
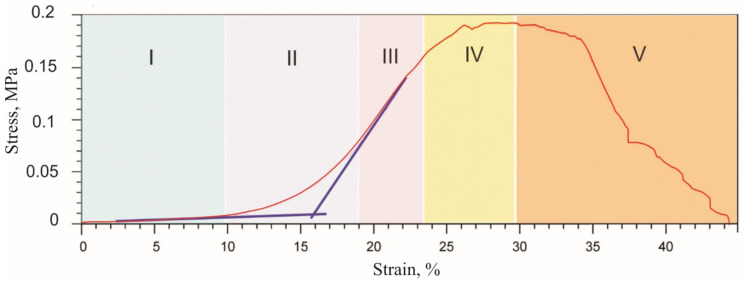
Engineering stress-strain curve of a single tension cycle before rupture of soft biological tissue. I—the initial linear region with low elastic modulus; II—the middle transition region of nonlinear deformation; III—the linear region with high elastic modulus; IV—yield region; V—rupture of the fibrous structure.

Biological tissues ruptured in a wide strain range during a single tension cycle, exhibiting several stages of fracture (Figure 5), which is characteristic of fibrous materials fracture.

Stress-strain curves with a softening effect, typical for viscous-superelastic materials, were obtained during the cyclic tension of biological tissues and a knitted mesh made from superelastic NiTi wire. Therefore, the obtained dependencies were analyzed from the superelastic material’s standpoint.

### 3.1. Rubber-Like Behavior of Skin, Muscles, and Tendons

**Figure 6:**
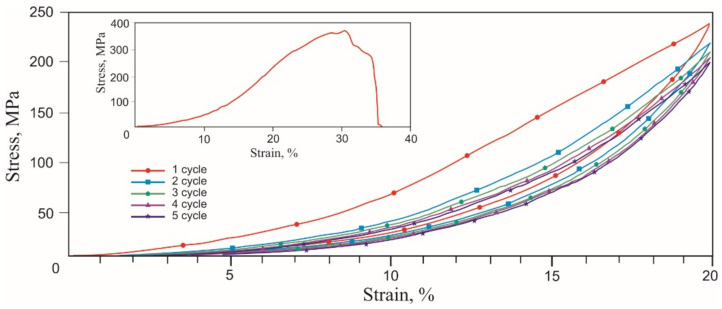
Engineering tensile stress-strain curves of the skin 5 loading-unloading cycles and uniaxial tension to rupture (inset).

**Figure 7:**
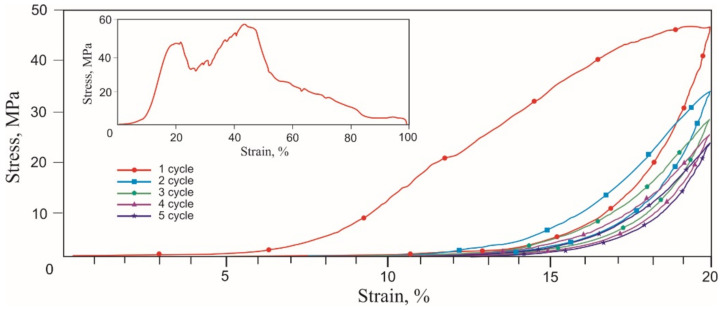
Engineering tensile stress-strain curves of the tendon 5 loading-unloading cycles and uniaxial tension to rupture (inset).

**Figure 8:**
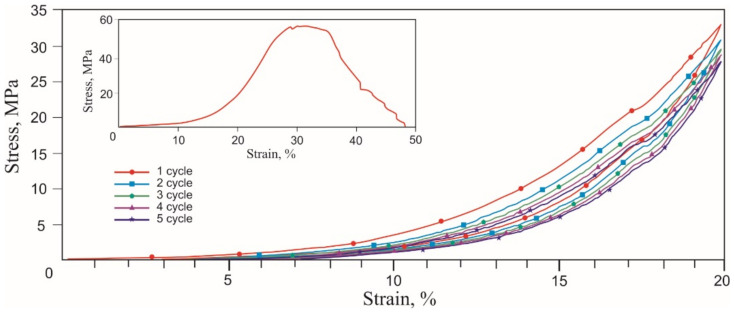
Engineering tensile stress-strain curves of the muscle in the 5 loading-unloading cycles and the uniaxial tension to rupture (inset).

Tensile stress-strain curves of the skin, tendons, and muscles were obtained in two stages. During the first stage of tension rupture, the transition areas of the elastic modulus increased, and the terminal areas were determined. During the second stage of cyclic tension at the transition region in the range of normal physiological loads, the Mullins-like effect was observed. The stress-strain curves of all biological samples looked similar qualitatively, exhibiting nonlinear stiffening effects, characteristic of superelastic materials (Figure 6, Figure 7 and Figure 8, Table 1).

A noticeable increase in the modulus of elasticity occurred in all tissues in the 6–20% strain range. The rupture of all tissues occurred at 30–40% engineering strain. Deviations of the characteristic elongation sections of the samples were associated with methodological errors in measuring the sample elongation during the preliminary tension of the samples before the beginning of the test. The tensile strength of the muscle (0.2 MPa) was lower than the tendon (0.4 MPa) and the skin (1.3 MPa). There was a high probability of a significant overestimation of the engineering tensile stress in the skin due to a large error in measuring the sample effective cross-section. The tensile strength of the tendon had intermediate values in the 0.35–0.45 MPa range. The appearance of two modes in the stress-strain curve was caused by the inhomogeneity of the tendon sample and the successive rupture of two large fibers. The different structure and density of skin, tendon, and muscle tissues caused differences in the tensile strength and the extent of fracture after reaching the tensile strength.

The similarity of the stress-strain curves of all tissues was manifested during cyclic tension. The Mullins softening effect was observed in all tissues, attributed to the uniaxial cyclic tension of superelastic materials and biological tissues.

During the second cycle, all samples showed a softening effect, expressed as a decrease in stress under load, typical of soft tissues [35,36]. At the unloading stage, the stress decreased significantly less. Both branches of the stress-strain curves tended to a minimum of stresses and a reduction in work against resistance forces when loaded and unloaded. The softening effect was more explicitly observed in the tendon, which has more collagen, and the least in the skin. The reason for the softening of biological tissues during cyclic tension is considered to be the selective deformation of the structural elements of biological tissues, which have a different modulus of elasticity. The counteraction of the forces of internal friction in the tissues leads to the delayed restoration of the shape after the removal of the external load. At the same time, structural elements that develop large elastic stresses recover their shape faster than elements with a lower elastic stress lag. Thus, there is an effect of the apparent softening in biological tissues. The effect of lowering the developed stresses is especially noticeable after the first loading cycle, but after the third cycle, the process of lowering the stresses slows down. The softening effect during cyclic stretching of tissues is the reason that some researchers deliberately do not take into account the results of the first 5–10 deformation cycles and call the first several cycles sample training.

During this sample training, the hysteresis area decreases and approaches a minimum. The area of the hysteresis loop graphically expresses the work against the viscous forces of internal friction in biological tissues. A decrease in its area due to the softening effect is a characteristic sign of the cyclic deformation of viscous-superelastic materials [15,36]. The transition region of the stress-strain curve is reduced to a minimum and becomes more pronounced after the first 1–3 cycles, accompanied by a decrease and stabilization of internal friction losses. The important features are the residual strain and the strain rate in the region of the low elastic modulus.

It is known that soft biological tissues are hyperelastic. The structural elements of soft tissues are formed of long molecular chains, which take on different configurations with similar internal energy, but different elastic moduli when stretched [37]. With the uniaxial tension of soft tissues, their configuration entropy decreases, and the elastic modulus increases. When the load is removed, the driving force to increase entropy returns the fibers to their original disordered state. This is entropic elasticity due to the ability of molecular chains to rotate around their single bonds, which makes it possible to obtain a large number of loop-like shapes, called conformations. The maximum entropy corresponds to the length of the molecular chain with the largest number of disordered conformations. The tensile force helps to straighten and lengthen molecular chains, reducing entropy in equilibrium. The force associated with entropy is determined from the thermodynamic relations [38]:

(2) A=U−TS

(3) dA=dW−SdT

(4) dA=fdL−SdT

(5) $$f={\left(\frac{dW}{dL}\right)}_{T,V}={\left(\frac{dA}{dL}\right)}_{T,V}$$

(6) $$f={\left(\frac{dA}{dL}\right)}_{T,V}={\left(\frac{dU}{dL}\right)}_{T,V}-T{\left(\frac{dS}{dL}\right)}_{T,V}=f_{U}+f_{S}$$

Here, *U* is the internal energy, *T* is the temperature in *K*, *S* is the entropy, *dW* is the work performed by external forces, *dA* is the increment of the Helmholtz free energy. Hence, the entropic elasticity is:

(7) $$f_{S}=-T\left(\frac{dS}{dL}\right)$$

Here, *dS*/*dL* is the rate of entropy change depending on the chain length. It is seen that the change in entropy is a configurational process. When soft biological tissues are loaded, a small instantaneous elastic deformation is accompanied by a much larger elastic deformation developing in time; thus, a state of delayed high elasticity is achieved.

### 3.2. Deformation of Titanium Nickelide Knitted Mesh and Superelastic Wire

**Figure 9:**
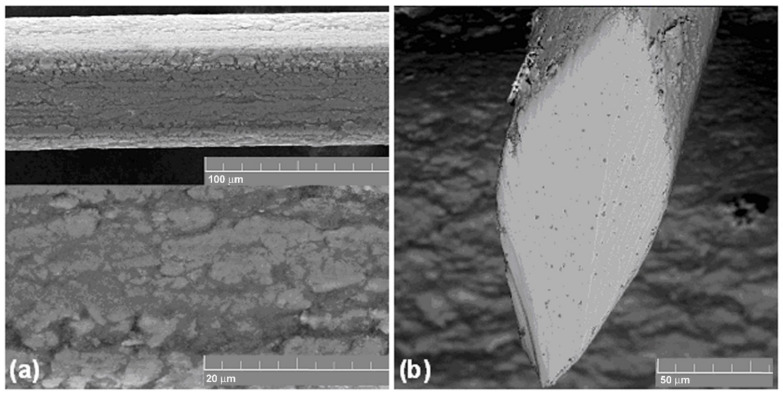
Superelastic 60 µm diameter NiTi wire: (**a**) side view; (**b**) cross-section.

A nickel-titanium wire less than 100 μm in diameter can be considered a composite material because at these dimensions the relative proportion of surface non-metallic phases and their effects on the physical, mechanical and electrochemical properties of the wire become significant [39,40]. The surface phases and the coatings are formed by the interaction of interstitial impurities with the surface layers of the NiTi matrix during cyclic plastic deformation in the presence of lubricants and annealing in the air (Figure 9). The interstitial impurities stimulate the matrix decomposition in the surface layers and titanium segregation to the surface. The decomposition products of the matrix react with interstitial impurities and form complex titanium oxycarbonitrides and titanium nickelide, resulting in a rough wire surface [39,40].

**Figure 10:**
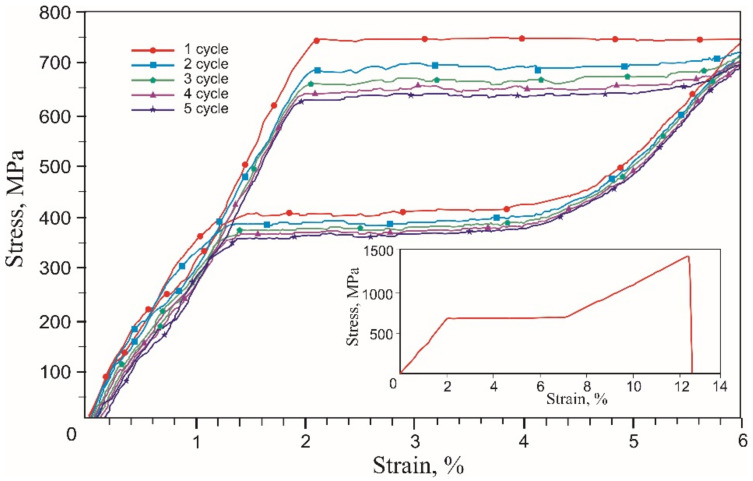
Engineering tensile stress-strain curves of the 60 µm NiTi wire 5 loading-unloading cycles and uniaxial tension to fracture (inset).

For comparison with the stress-strain curves of mesh knitted from NiTi wire, 60 μm diameter NiTi wire was tested. The obtained engineering stress-strain curves of the NiTi wire corresponded to typical stress-strain curves of nitinol wire (Figure 10).

The uniaxial tension stress-strain curve to fracture had three linear regions: B2 austenite elastic deformation up to 2% strain, viscous flow region at 2–7.5% strain associated with the direct martensitic transformation of austenite into martensite B2 → B2 + R → B19^/^ and linear hardening associated with B19^/^ martensite deformation. In the elastic deformation region of austenite at a critical stress level, austenite becomes unstable, and the nuclei of the stress-induced martensite phase begin to form. Upon reaching the critical martensitic shear stress, martensite propagates in the sample under constant stress and forms a region of viscous flow in the form of a plateau associated with the growth of martensite bands [41]. The result of this phase transformation was a 5.5% change in strain. The yield point coincided with the elastic limit of 700 MPa. The wire 1500 MPa tensile strength was achieved at 13% strain.

**Figure 11:**
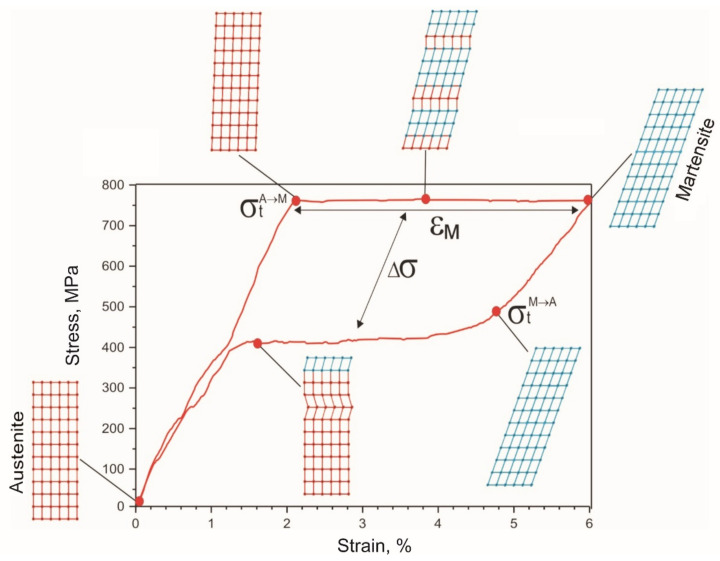
Schematics of twinning in characteristic regions of the NiTi wire uniaxial tension stress-strain curve.

A shape memory alloy wire undergoing a stress-induced martensite transformation during a loading-unloading cycle exhibited superelastic behavior (Figure 11). The value of the critical stress of the forward martensitic transformation σ_t_ was 780 MPa during the first tensile cycle. In the stretching process, 4% of inelastic martensitic deformation ε_M_ was fully recovered with a delay during unloading, forming a mechanical hysteresis Δσ of 360 MPa. The area of the superelastic hysteresis curve corresponded to the dissipated mechanical energy due to internal friction during the movement of the austenite-martensite interface.

**Figure 12:**
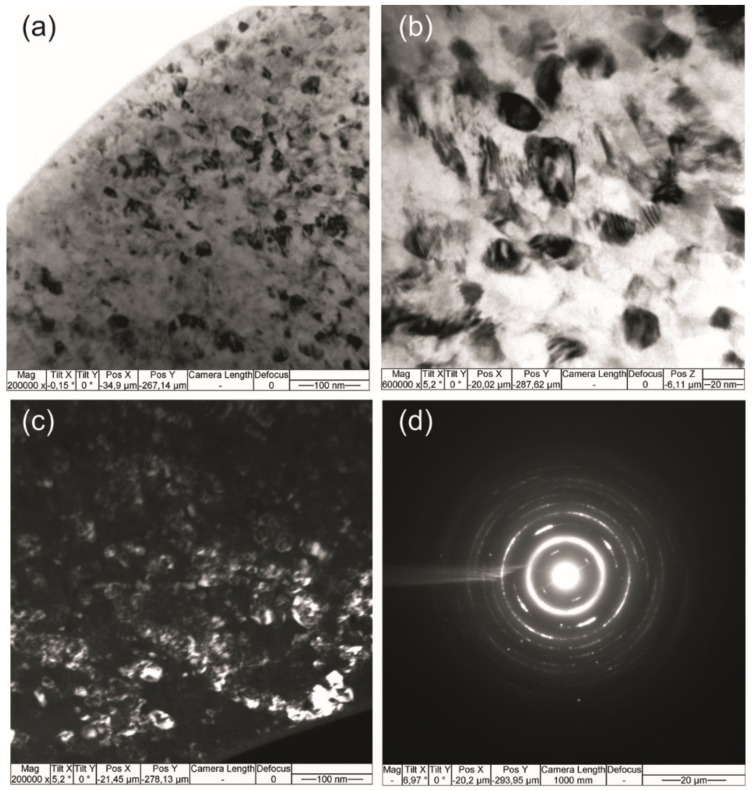
(**a**–**c**) TEM images of the nanocrystalline structure of 60 μm NiTi wire and (**d**) the corresponding diffraction pattern from the B2 phase.

The absence of residual strain was due to the uniform nanocrystalline structure of the wire with an average grain size of 20 nm. The bright-field TEM image showed a grain structure represented by nanograins of the B2 phase (Figure 12a,b). A deformation contrast was found inside the grains, which was enhanced near the grain boundaries. In particular, there was a Moiré pattern (interference contrast) due to the superposition of diffraction effects from grains with close interplanar spacing. The sources of stresses in grains were the incoherent boundary and misfit dislocations that created low-angle boundaries. The grain structure was not recrystallized, as evidenced by the inhomogeneous background of the extinction contours. Fragments of the subgrain structure, depicted in a dark field, are no larger than 10 nm. The microdiffraction showed texture defects associated with the azimuthal smearing of diffraction reflections along each of the planes of the B2 phase, especially along the (100) plane (Figure 12c). In some areas, there were accumulations of particles elongated in one direction due to the drawing process. The dark-field TEM images showed grains from the (100) reflection corresponding to the B2-NiTi austenite phase (Figure 12d).

Thus, dislocations and grain boundaries acted as centers of heterogeneous nucleation of martensite crystals [42,43]. This was in agreement with experimental results, showing that grain boundaries in NiTi could promote the superelastic martensitic transformation.

It should be noted that, in this case, the small grain size did not suppress the superelastic behavior, in contrast to [44,45,46], where the superelasticity strongly depended on the grain size, and at a grain size less than 50 nm in NiTi, the martensitic transformation was completely suppressed.

Single and cyclic uniaxial tensile tests of NiTi mesh knitted from 40 μm, 60 μm, and 90 µm diameter wires were carried out. It was extremely important that, under a single uniaxial tension to fracture, the stress-strain curves did not show any yield areas caused by the martensitic transformation, which were found in the uniaxial tension stress-strain curve of a single wire. This indicated that the stresses in most of the knitted NiTi mesh stretched by 10% did not reach the martensitic shear stress and remained below the elastic limit.

During the single uniaxial tension of the knitted 60 μm NiTi mesh, it was deformed elastically with a constant elastic modulus up to an initial 20% strain. At a 20–40% strain, the elastic modulus increased nonlinearly, and at a 40–50% strain, it became constant again, but much larger than in the initial loading. In the final 50–55% strain range, the modulus again decreased nonlinearly. The elastic moduli of the knitted 40 µm and 90 μm NiTi mesh changed similarly.

The tensile strength of the 40 μm knitted mesh was commensurate with the tendon but exceeded the tensile strength of the skin and muscles. The tensile strength of knitted 60 μm and 90 µm mesh exceeded biological tissues. Increasing or decreasing the wire diameter could significantly change the tensile strength of the knitted mesh. The fracture of the knitted mesh was viscous-brittle since at the final 5% stage of the stress-strain curve, the hardening plateau was reached and a brittle fracture occurred. In the contact areas of the mesh loops, the tensile strength of the wire was quickly and simultaneously reached due to the rapid hardening of the alloy in the martensitic state, which was characterized by the brittle fracture.

**Figure 13:**
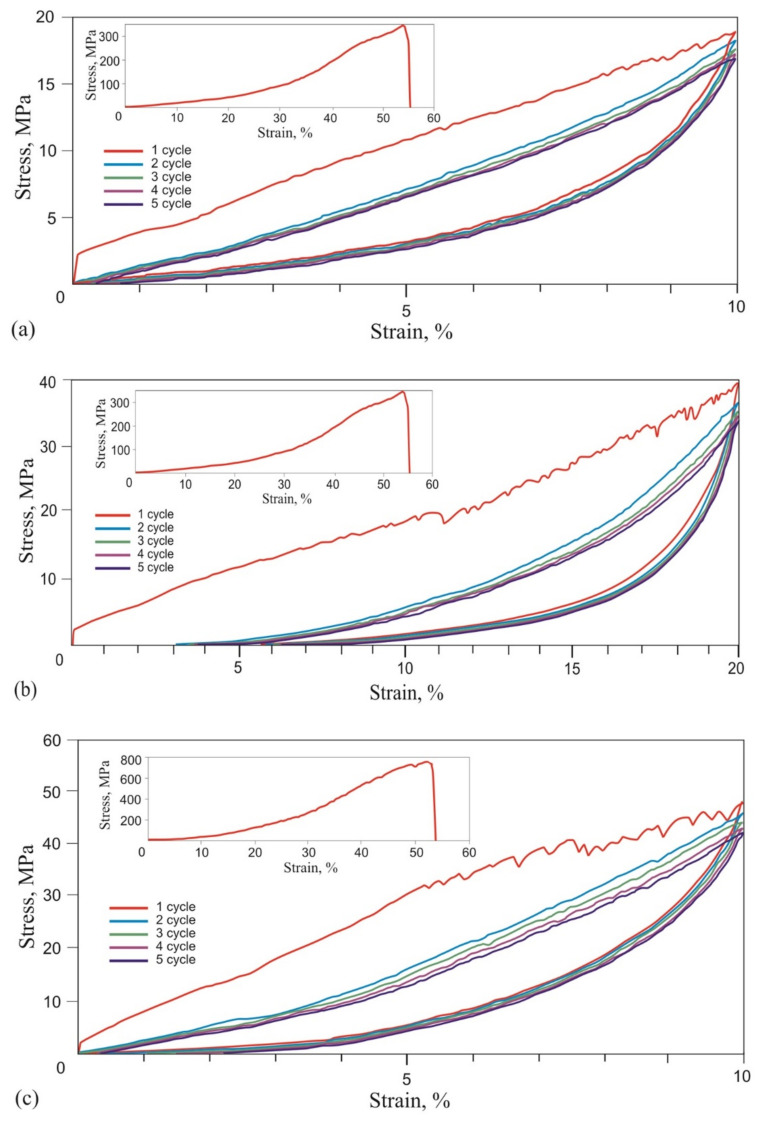

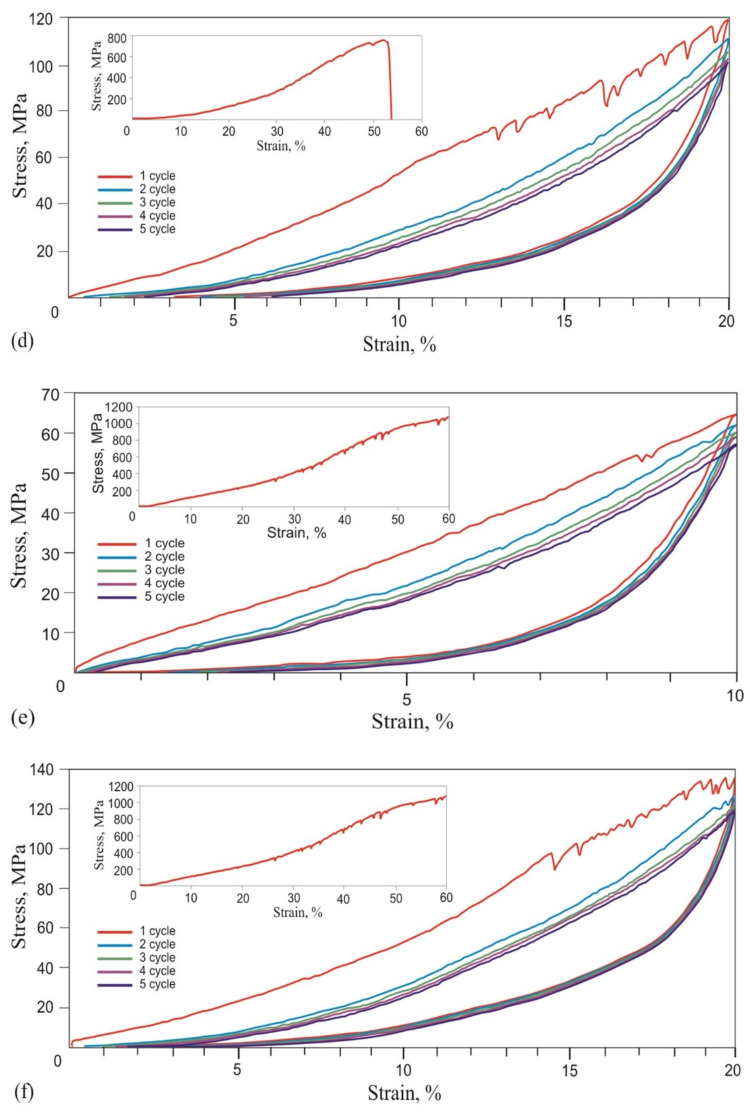
Engineering stress-strain curves of (**a**,**b**) 40 µm, (**c**,**d**) 60 µm, and (**e**,**f**) 90 µm knitted NiTi mesh under 5 loading-unloading cycles; uniaxial tension to fracture (insets).

Tensile stress-strain curves were obtained for single and cyclic uniaxial tensions of the knitted NiTi mesh by 10% and 20%, similar to soft biological tissues and characteristic of superplastic materials (Figure 13). They were similar to the stress-strain curves of the skin. In all cases of cyclic uniaxial tension after the first loading cycle, the hysteresis loop area decreased and stabilized in Figure 13.

Cyclic uniaxial tensile tests were carried out within the normal physiological strain of 10% and did not reach the critical elongation at which elastic deformation transformed into plastic. The elongation depended on the thickness of the wire and the design of the knitted mesh. The areas of the knitted mesh that were outside the contact areas were elastically deformed by no more than 2%. Linear areas with a low and high modulus of elasticity were found in all stress-strain curves. This was seen in the 40 μm wire cyclic tension stress-strain curves with a 20% strain. In this case, the residual strain of 3% was visible.

Cyclic 10% stress-strain curves were linear during loading and lost linearity during unloading. The modulus of elasticity and maximum stresses decreased with each subsequent tension cycle. This softening effect in superelastic materials is called the Mullins effect. The softening effect during the cyclic tension of the knitted mesh was due to the ratio of the elastic forces developed in the loops during unloading and the viscous frictional resistance between the loops.

At the first unloading cycle of the knitted mesh, elastic regions of the stress-strain curves were present, associated with loaded regions of the knitted mesh loops. In this case, the elastic restoring forces with a high modulus overcame the forces of internal friction more effectively than low modulus ones, and, thus, the effects of softening and delayed unloading were manifested. Frictional forces prevented low-modulus elastic forces, which tended to return the loops to their original state, and the knitted mesh acquired an optimal stable configuration.

At a 20% tension of the knitted mesh, a decrease in the thickness of the wire and a decrease in the elastic restoring forces led to a greater loss to the friction forces. As a result, the residual strain of the 40 μm knitted NiTi mesh was 3%. In this case, the elastic tensile deformation also became nonlinear for all diameters ranging from 40 μm to 90 μm and corresponded to the model of superelastic tension. At a 40 μm diameter, nonlinearity was noticeable even in the first tension cycle.

At the initial moment of the first tension cycle of the 40 μm and 60 μm knitted NiTi mesh, a stress increase with an extremely high modulus of elasticity was detected. This was due to the dissipative process of overcoming the static friction between the loops. This effect was more pronounced with 40 μm than with 60 μm and 90 μm wires. After the first deformation cycle, the viscoelastic flexible system stabilized and assumed the optimal configuration.

Observed dynamic hysteresis was characteristic of cyclically loaded viscoelastic systems. The hysteresis area of the 1st cycle significantly exceeded the area of the 2nd and subsequent cycles (Figure 4a). The first cycle was associated with an irreversible shift of the loops, and the second and subsequent cycles were associated with a reversible slip of the loops relative to each other. During the first cycle, the knitted loops overcame the friction at rest and took an optimal position, which was maintained during subsequent cycles. Therefore, the second region was more stable than the first. The energy to overcome friction forces was almost half after the first cycle. The difference in areas between the 1st and the following cycles’ hysteresis increased markedly with a decrease in the wire diameter and an increase in elongation up to 20%. This meant an increase in the viscous drag force in the ratio of elastic and viscous forces.

The shape of the hysteresis loop in the first loading cycle of all knitted NiTi meshes differed from the following ones, but the shape of the loops from the 2nd to the 5th cycles was very similar, which showed a tendency to stabilize after the first loading-unloading cycle. The area of the hysteresis loop in the first cycle was much larger than in the following cycles.

In the knitted mesh, stresses were distributed extremely unevenly. Therefore, with a uniaxial tension of the knitted mesh to fracture at the contact areas of the loops, the stresses reached not only the ultimate martensitic strain but also the ultimate strength. Under cyclic tension in the elastic range, no signs of plastic deformation were found in the contact areas. In the unconstrained areas of the loops, the stresses did not cause a martensitic transformation, since the tensile deformation remained linear without residual strain at 10% tension and had a slight nonlinearity and 3% residual strain at 20% tension.

Consequently, part of the heat from internal friction during the martensitic transformation was released locally at the points of contact of the loops, while the main heat was released as a result of surface friction between the loops of the knitted mesh. Thus, as a result of the uneven distribution of the load in the knitted loops, the effect of superelasticity could manifest itself only locally at the contact areas of the loops. The rest of the loops experienced only elastic deformation, and the knitwear as a whole behaved as a viscous-superelastic material [15,47].

Several common features were found in the cyclic tensile stress-strain curves of skin, tendons, muscles, and the knitted NiTi mesh (Figure 6, Figure 7, Figure 8 and Figure 13). The uniaxial tension stress-strain curves for all samples were superelastic or rubberlike. All samples under cyclic tension showed the softening and delay effects with low and high-modulus regions distinguished in each stress-strain curve. Stresses, the elastic modulus, and dynamic hysteresis of all samples were stabilized by the 3rd tension cycle. In this case, the low-modulus region of elastic deformation during unloading became stable from the first cycles.

Several differences between the cyclic tension of the knitted NiTi mesh and soft tissues were also found. The most noticeable feature of knitted mesh deformation was the linear nature of the elastic deformation at 10% elongation and the complete absence of residual strain during unloading. The softening effect during cyclic tension of the knitted mesh was not as pronounced as in biological tissues. The skin and knitted mesh stress-strain curves were the closest.

A knitted mesh, similar to soft biological tissues, is not a solid object, but a tensegrity structure, the loops of which are constrained, but retain individual mobility. The tensegrity structure deformed extremely non-uniformly. The contact sections of the loops experienced extreme bending, at which the stresses approached the ultimate strength, while the remaining sections experienced tension and bending at stresses well below the yield stress. Therefore, elastic deformation in different sections of the loops relaxed at different rates.

Considering that during the cyclic tension of the knitted NiTi mesh there was a variable residual strain and stress hysteresis, depending on the wire diameter, it can be assumed that the losses were associated with friction at the contact areas during slipping of the loops. It is known that the Mullins effect is characteristic of tensegrity structures [48], rubber-like materials [49], cell membranes [50], and pressed shape-memory wires [51]. Comparing the stress-strain curves of a 60 μm wire and a knitted mesh created from it, it is clear that the tensegrity structure of the knitted mesh limited the manifestation of the superelastic effect inherent in the wire.

The authors’ experience of using a knitted NiTi mesh in animal experiments, where it withstood more than 17 million cycles of a physiological respiratory load without fracture, allowed asserting that titanium nickelide wire under high-cycle loading was capable of withstanding up to 6% relative elongation without fracture, accumulating no more than a 0.3% residual strain.

The loading region remained linear for all cycles at 10% tension but became non-linear after the first cycle at 20% tension. This behavior suggests that, for a given knitted mesh design, an elongation of less than 20% was insufficient to overcome Hooke’s region of elastic deformation.

The results of cyclic loading of knitted mesh were used in an in vivo study to test the quality of knitted mesh integration into soft biological tissues. For this purpose, a 60 μm knitted NiTi mesh was chosen since with a minimum pre-tension, it showed a minimum softening effect and a minimum decrease in stress hysteresis after the first cycle of physiological deformation of 6%. This deformation behavior is important for the frame function of the implant in reconstructive surgery of abdominal wall defects.

### 3.3. Knitted Mesh Implants Biocompatibility in Laboratory Animals

The study was carried out by replacing the thoracoabdominal defect in the anterior abdominal wall of rats. The elastic properties of the titanium nickelide knitted implant and the concerned structures of the chest and abdominal walls are similar. Therefore, under a physiological load, the formed tissue-implant complex was deformed coherently. The peculiarities of the implant fixation made it possible to reliably fix the mesh implant along the edges of the defect and evenly distribute the load over the contacting surfaces of the implant and tissues.

No migration of the implants or postoperative complications were observed. Morphological studies of the surgical intervention area in animals indicated the formation of similar structure tissue, which regenerated in the replaced thoracoabdominal area.

**Figure 14:**
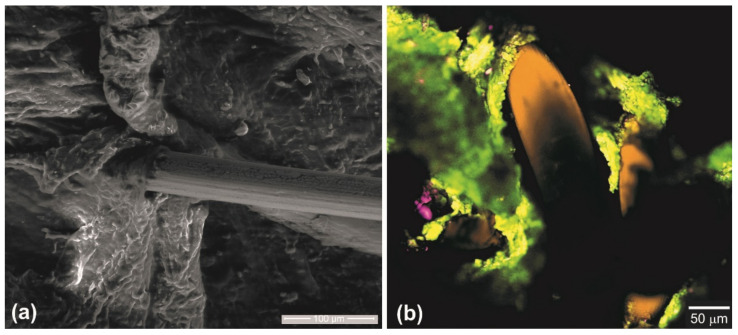
Knitted mesh from 60 µm NiTi wire integrated into the rat tissues of the anterior abdominal wall: (**a**) SEM; (**b**) confocal microscopy.

No significant changes that could lead to the disruption of the organ’s function were found in the adjacent organs of the abdominal cavity. The implant was fixed to the chest wall and the diaphragm through a dense, but non-coarse, connective tissue regenerate with a small number of cellular elements and a characteristic orientation of connective tissue bundles along the implant surface. The regenerate was positioned along the free edge of the implant in the form of a sleeve. The newly formed tissue grew through the mesh implant with the formation of a single tissue regenerate in the defect zone, which provided an anatomical and physiological restoration of this area (Figure 14).

## 4. Conclusions

1. A 60 μm diameter NiTi alloy wire reached martensitic transformation stress of 750 MPa during five loading-unloading cycles and exhibited the superelasticity effect at a 6% engineering strain. Wire rupture in the 1450–1500 MPa range had brittle fracture features.
2. During the single loading of the mesh made from the NiTi wire, up to the tensile strength, and during cyclic loading up to 10% and 20% relative strain, the yield strength caused by martensitic transformation and the NiTi superelasticity effects were not found. The rupture of metal mesh in the 350–800 MPa range also exhibited a brittle fracture.
3. The cyclic tension stress-strain curves of the metal mesh made from NiTi wire exhibited superelastic behavior, reversibly changing the structure under the action of external loads. A characteristic feature of superelastic behavior is the observed softening effect. The residual macro deformation of the mesh after the first two tensile cycles was due to the interaction between the contact sections of the loops: slip under load and friction, which counteracted the elastic unloading.
4. The comparative analysis of cyclic loading showed that the deformation behavior of the mesh made from 40 µm, 60 µm, and 90 µm NiTi wire was similar to the superelastic behavior of skin, tendons, and muscles.
5. In addition, in the knitted NiTi mesh, as well as in soft biological tissues, the effects of softening and delayed elastic unloading were observed. This effect was due to the variable modulus of elasticity of the loops, due to the inhomogeneity of the distribution of elastic stresses in the knitwear loops, the viscous slip of the loops, and viscous friction, which prevented the elastic deformation of the loops.
6. An in vivo clinical experiment showed good integration of a superelastic knitted NiTi wire mesh into living biological tissues under normal physiological stress. Due to the similarity of the deformation behavior of the structures of the chest and abdominal walls and the mesh implant, the formed tissue-implant complex was deformed in concert, and the load was evenly distributed over the implant-biological tissue interface.
7. The observed similarity of the knitted mesh stress-strain curves for all wire diameters and in the entire range of stresses and strains suggested that there was a possibility of choosing a knitted NiTi mesh with the required strength and deformation characteristics for different types of soft tissues. The main criteria for the rheological similarity of the knitted NiTi mesh and soft tissue were tensile strength, deformation range of low and high elastic moduli during loading and unloading, the value of the elastic moduli in the loading and unloading zones, and the amount of permanent deformation during the cyclic stretching of metal knitwear. Further development of the proposed methods for comparing biomechanical properties will make it possible to develop objective criteria for choosing a knitted NiTi mesh for reconstructive and organ-preserving soft tissue surgery.

## Figures and Tables

**Table 1:** Mechanical properties of different tissues.

Type of Tissue	Skin	Tendon	Muscle
Tensile strength, MPa	1.4	0.4	0.2
Fracture region, %	27–33	17–92	27–45
Middle transitional region of nonlinear deformation, %	9–12/7–8/12–13	6–9/6–9/15–17	13–17
Maximum stress during cyclic loading, MPa	0.95	0.27	0.08
Minimum stress during cyclic unloading, MPa	0.01	0.01	0.04

## Data Availability

All data are presented in the paper.
